# Identification of population characteristics through implementation of the Comprehensive Diabetic Retinopathy Program

**DOI:** 10.1186/s40842-019-0079-6

**Published:** 2019-05-09

**Authors:** Nish Patel, Lilia Verchinina, Michele Wichorek, Thomas W. Gardner, Dorene Markel, Jennifer Wyckoff, Anjali R. Shah

**Affiliations:** 1 University of Michigan Medical School, Ann Arbor, MI USA; 2 The Brehm Center, University of Michigan, Ann Arbor, MI USA; 3 Kellogg Eye Center, Department of Ophthalmology and Visual Sciences, Michigan Medicine, Ann Arbor, MI USA; 4 Molecular and Integrative Physiology, University of Michigan, Ann Arbor, MI USA; 5 Learning Health Sciences, University of Michigan Medical School, Ann Arbor, MI USA; 6 Metabolism, Endocrinology, and Diabetes, Michigan Medicine, Ann Arbor, MI USA

**Keywords:** Diabetic retinopathy, Diabetes, Characteristics, Program, Longitudinal, Risk, HbA_1c_, Blood pressure

## Abstract

**Background:**

Diabetic retinopathy is the most common cause of blindness in working-age adults. Characteristics of patients with diabetes presenting to a retina subspecialty clinic have not been adequately studied, limiting our ability to risk stratify patients with diabetic retinopathy. Our goal is to describe an innovative program that collects structured, longitudinal data on patients with diabetes in a retina clinic, and identifies population characteristics to define patient risk stratification.

**Methods:**

Demographics, body-mass index, blood pressure, hemoglobin A_1c_, smoking history, diabetes type, diabetes duration, kidney disease history, and diagnosis codes were collected on all patients with diabetes at the Kellogg Eye Center retina clinic. A mixed effects negative binomial regression was then performed to assess visit frequency as a function of these variables. Visit frequency was used as a marker for cost of care. A subgroup of patients was surveyed about knowledge of diabetes management goals and barriers to better self-management.

**Results:**

There were 2916 patients in the cohort with 1014 in the subgroup. The cohort was predominantly Caucasian (74.5%), with a mean age of 64 years (range 13–99) and a relatively even distribution of sex (53.2% men). The mean maximum hemoglobin A_1c_ was 8.0% (range 4.3–15.7%), and 57.1% had a diagnosis of diabetic retinopathy. Patients averaged 3.9 visits (range 1–27) during the 18-month study period. Blood pressure and duration of diabetes were positively associated with visit frequency (*p* < 0.0001, p < 0.0001, respectively). Of the surveyed patients, 87.6% knew their goal hemoglobin A_1c_, while only 45.9% identified the correct blood pressure goal. The most common reported barrier to better self-management was “it’s just not working” (47.1%).

**Conclusions:**

Implementation of this program enables the creation of a longitudinal dataset on patients with diabetes. Resulting data can be used to improve quality of care provided to patients at a retina clinic. The findings suggest considerable healthcare resources are being directed to a small patient population. This enhanced understanding of characteristics of patients with diabetes will improve efforts to preserve vision and utilize health system resources efficiently.

## Background

Diabetic retinopathy (DR) is one of the most common complications of diabetes mellitus [1] and is the leading cause of blindness in working-age adults in the world [2], with the number of affected individuals worldwide expected to increase from 126 million in 2010 to 191 million by 2030 [2]. In addition, DR represents a significant financial burden to society. In the United States, the direct medical costs alone for DR were estimated at more than $490 million in 2004 [3]. This, however, is likely an under-estimation of the current direct costs, as treatment of DR has changed significantly since 2004 with the introduction of intravitreal anti-vascular endothelial growth factor agents. Unfortunately, more recent estimates of DR costs are not available. In addition, indirect costs, such as job absenteeism and burden on family members, are not included. There is also little understanding of which patients are most likely to develop vision-threatening DR and require treatment, versus who will not. With dramatic projected increases in the number of persons with diabetes and associated costs of their care, it will be critical to improve the efficiency of health care resource use in the future. In this report, we describe a novel program designed to collect systemic health data in the setting of an ophthalmology clinic, with the goal of improving care and resource management in the future.

Considerable research has elucidated the risk factors for and the effect of interventions on the development and progression of DR. Longer duration of diabetes mellitus, poor glycemic control as measured by hemoglobin A_1c_ (HbA_1c_), and higher blood pressures (BPs) are associated with increased incidence and prevalence of DR [4–8]. Increased HbA_1c_ and longer diabetes duration, in particular, are consistently shown to also be associated with progression to more severe retinopathy [6, 8, 9].

Fortunately, aggressive control of both blood glucose and BP significantly reduces the risk for development and progression of DR [10–17]. Interestingly, the United Kingdom Prospective Diabetes Study follow-up studies in 2008 showed that the beneficial effects of intensive glycemic control on any diabetes-related end point, microvascular disease, myocardial infarction, and all-cause mortality in patients with type 2 diabetes were sustained for up to ten years after cessation of interventions while similar benefits of BP control were lost at the ten-year mark [11, 12].

Despite the clear importance of parameters such as HbA_1c_, and BP in the prevention and treatment of DR, it is not standard practice for ophthalmologists to actively check HbA_1c_ or BP during routine eye care visits, even if these values are otherwise unavailable from the patient record. Therefore, many ophthalmologists are unaware of their patients’ overall diabetes management status. This lack of awareness is surprising given the frequency with which patients with retinopathy see ophthalmologists. Management of DR often involves intraocular procedures, frequently performed on a monthly basis, which can put significant strain on the patient and family, as well as health care resources. Yet, it is unclear which patients will progress to requiring treatment, and which will need aggressive management. Without adequate data on systemic diabetes management parameters, ophthalmologists are unlikely to be able to develop risk algorithms or to improve prognostic capability. The purpose of this study is to report on the novel implementation of a sustainable program that combines survey data, electronic health record (EHR) data, and when otherwise unavailable, screening data obtained during routine ophthalmology clinic visits, and to report on initial findings from analysis of this data. A more comprehensive understanding of patients’ diabetes status will allow ophthalmologists to better risk stratify patients and tailor care accordingly, leading to a more efficient use of health care resources. In addition, a more thorough understanding of DR in the context of systemic diabetes care will allow for future quality benchmarking and improvement.

There are few reports describing systemic characteristics of patients with diabetes presenting to a retina or eye clinic. One such study, conducted in 1998, evaluated 118 patients with diabetes who newly presented to an inner city public eye hospital in Los Angeles. The patient population consisted predominately of minority groups (55% Hispanic and 43% African American) and individuals of low socioeconomic status [18]. This study did not examine HbA_1c_ or BP. These data are unlikely to represent diabetic populations today, since the management of systemic diabetes and DR have changed significantly in the past two decades. In addition, the relatively small sample size and study location limit potential extrapolation.

To our knowledge, a program designed to routinely collect systemic health data on patients with diabetes presenting to a retina clinic has not been previously implemented.

## Methods

The Comprehensive Diabetic Retinopathy Program (CDRP) was developed by the faculty from the Michigan Medicine Ophthalmology and Visual Sciences retina clinic, the Metabolism, Endocrinology and Diabetes Clinic and the University of Michigan Brehm Diabetes Center, in conjunction with current American Diabetes Association guidelines. CDRP was developed as a quality improvement project, and was thus IRB-exempt. The goal of the program was to incorporate data acquisition, including point-of-care HbA_1c_ testing and BP measurements into normal retina clinic workflow, in the hopes that future analysis of this data will impact patient management decisions. In addition, all relevant diabetes information is stored in a structured manner, such that it is easily extracted for analysis, while also being available to the physician as part of an easily accessible “form” linked to each individual patient encounter in the EHR.

Demographics, body mass index (BMI), BP, HbA_1c_, smoking history, diabetes type, diabetes duration, kidney disease history based on both self-report and microalbuminuria (≥ 30 μg albumin/mg creatinine), and diagnosis codes were collected on all patients with diabetes presenting to the Kellogg Eye Center retina clinic for new patient visits and return visits from July 1, 2016 to December 30, 2017 via survey questions and information available in the EHR. Microalbumin was chosen as a marker because it is a screening test shown to be highly sensitive for early diabetic nephropathy [19], and because it is used as a marker by the diabetes quality improvement group at the institution. Though values were recorded for each patient at each encounter, point-of-care HbA_1c_ and BP were only checked in the clinic if no values were already present in the EHR from the previous six months. For patients who had multiple visits and correspondingly multiple measures, the maximum value has been used for the analysis. The number of visits is calculated only for those patients who presented to the clinic for at least 12 months. To analyze visit frequency, we ran a mixed effects negative binomial regression with the number of visits as an outcome and age, sex, race, maximum HbA_1c_, presence or absence of DR diagnosis codes, the interaction term between maximum HbA_1c_ and DR diagnosis codes, diabetes type, duration of diabetes for ≥10 years, kidney disease history, maximum systolic blood pressure (SBP), maximum diastolic blood pressure (DBP) as fixed effects and patients’ providers as a random effect. Mixed effects negative binomial regression as well as frequencies, mean, standard deviations and ranges have been calculated using SAS (version 9.4, SAS Institute, Triangle Park, NC, USA).

Ophthalmic technicians obtained point-of-care HbA_1c_ and BP measurements on all patients identified as having diabetes who did not have values from within 6 months in the EHR, regardless of whether or not they were being seen for diabetic retinopathy. In addition, these patients were asked about their knowledge of any history of kidney disease, and whether they had diabetes for over 10 years. Other information was obtained from pre-existing data in the EHR.

A subgroup of patients with 2 or more of the following factors, determined “high risk” for complications by endocrinologists based on previously published data [6, 20–22] and clinical experience, was targeted for additional data collection and analysis: HbA_1c_ > 9% or BP > 140/90 mmHg in the last six months, history of kidney disease based on either self-report or microalbuminuria, diabetes duration ≥10 years. From July 1, 2016 to July 1, 2017, subgroup patients were asked to complete a three-question multiple-choice survey assessing knowledge of diabetes management goals. During the entire study period, subgroup patients were also verbally surveyed about their perceived barriers to better control of their diabetes. In addition, they were provided literature on other community resources, such as smoking cessation and diabetes education classes. Technicians conducting these surveys and providing this additional information had completed 10 h of standard patient diabetes education classes and undergone a two-hour training session with an endocrinologist to ensure their comfort with the material being discussed.

## Results

Data were recorded from 2916 unique patients with 8470 encounters (Table 1). The following was the racial composition of the cohort: 74.5% white/Caucasian, 14.8% black/African American, 4.6% Asian, and 6.2% unknown/other/mixed. 64.6% of patients had type 2 diabetes, and there was a relatively even distribution of sex (46.8% women and 53.2% men). The mean age was 64 years (range 13–99), and the cohort was predominantly obese, with mean maximum BMI 33.5 kg/m^2^ (SD = 7.96). Mean maximum HbA_1c_ was 8.0% (range 4.3–15.7%), with the mode of the distribution at the value of 7.2% with a tail toward larger values; 66.2% of patients had HbA_1c_ values > 7 and 22.6% of patients > 9%. Although data were acquired on every patient with diabetes presenting to the retina clinic, only 57.1% (1664) had any encounter diagnosis of DR; other patients were being seen, presumably, for other problems. The average number of visits was 3.9 in an 18-month period (range 1–27 visits).

**Table 1:** Characteristics of Patients with Diabetes Presenting to a Retina Clinic

	*Entire cohort*				*Subsample: risk score≧ 2*			
	*N = 2916*				*N = 1014*			
	*N or Mean*	*% or SD*	*Min*	*Max*	*N or Mean*	*% or SD*	*Min*	*Max*
Patient age Mean (SD)	64.05	14.29	13	99	62.79	13.89	17	98
Race N(%)								
Asian	134	4.60			39	3.85		
Black, African American	431	14.78			198	19.53		
White or Caucasian	2171	74.45			715	70.51		
Unknown/other/mixed	180	6.17			62	6.11		
Sex N(%)								
Female	1365	46.81			467	46.06		
Male	1551	53.19			547	53.94		
Ever smoked N (%)								
Yes	1287	44.14			415	40.93		
No/unknown	1629	55.86			599	59.07		
Diabetic retinopathy N (%)								
Yes	1664	57.06			806	79.49		
No	1252	42.94			208	20.51		
Diabetes type N(%)								
Type 1	356	12.21			143	14.10		
Type 2	1883	64.57			736	72.58		
Undefined	677	23.22			135	13.31		
Maximum microalbumin N(%)								
< 30	239	8.20			96	9.47		
≧30	186	6.38			159	15.68		
Unknown	2491	85.43			759	74.85		
History of kidney diagnosis N(%)								
Yes	509	17.46			436	43.00		
No/unknown	2407	82.54			578	57.00		
Duration of diabetes ≧10 years								
Yes	1404	48.15			885	87.28		
No	1512	51.85			129	12.72		
Max patient’s BMI Mean (SD	33.46	7.96	17	81	34.02	8.39	17	81
Maximum systolic blood pressure Mean (SD)	141.91	22.86	80	237	152.71	22.7	87	237
Maximum diastolic blood pressure Mean (SD)	73.73	11.53	40	123	77.19	11.83	42	118
Maximum HbA_1c_ Mean (SD)	8.01	1.80	4.30	15.70	8.68	2.01	4.70	15.70
Patient’s number of visits Mean (SD)^a^	3.91	3.70	1	27	4.85	4.11	1	25

^a^For the number of visits outcome a subcohort of patients who presented with at least 12 months of potential follow up has been evaluated (*N* = 1504 for the whole cohort; *N* = 634 for the subgroup of patients with risk score ≥ 2)

A mixed effects negative binomial regression model identified variables associated with number of visits to the retina clinic (Table 2). Only patients who presented with the potential for at least 12 months of follow up were included in the analysis of total number of visits. In our sample, missing HbA_1c_ and BP values were more common in patients without a diagnosis of DR (of any kind). To control for this non-random factor of missing values, we included an indicator for presence of DR in our model. Random effect was also statistically significant (*p* < 0.001). The interaction term between patient’s maximum HbA_1c_ and DR was statistically significant (*p* = 0.006). Among patients with a diagnosis of DR, HbA_1c_ was not statistically significantly associated with the number of visits while controlling for other covariates. Other variables that were positively associated with visit frequency include Caucasian race versus Asian (*p* = 0.048), unknown type of diabetes versus type 2 (< 0.0001), SBP (< 0.0001), DBP (< 0.0001), and a duration of diabetes ≥10 years. Type 1 diabetes vs. type 2 (< 0.0001) was negatively associated with more frequent follow-up while holding other covariates constant. The magnitude of the scaled Pearson statistic (0.95) indicates that there was no over-dispersion in our model.

**Table 2:** Negative binomial mixed^a^ effects regression: Association between number of visits and patient characteristics

	*Cohort: visited the clinic for at least 12 months*			
	*n = 963*			
*Effect*	*Estimate*	*Standard Error*	*t Value*	*Pr > |t|*
Age	0.00007	0.002	0.04	0.969
Female (ref = Male)	0.03	0.04	0.75	0.454
Race				
Black or African American (ref = Asian)	−0.03	0.11	−0.28	0.776
Unknown/other/mixed (ref = Asian)	0.03	0.13	0.22	0.826
White or Caucasian (ref = Asian)^b^	0.20	0.10	1.98	0.048
Diabetes type				
Type 1 (ref = Type 2)^b^	−0.40	0.08	−5.26	<.0001
Undefined (ref = Type 2)^b^	0.21	0.06	3.28	0.001
Duration of diabetes ≧10 years (ref < 10 years)^b^	0.18	0.05	3.56	0.0004
History of kidney diagnosis Yes (ref = No)	0.08	0.05	1.74	0.082
Maximum systolic blood pressure (scaled by 10)^b^	0.05	0.01	4.38	<.0001
Maximum diastolic blood pressure (scaled by 10)^b^	0.16	0.03	6.00	<.0001
Maximum HbA_1c_^b^	−0.08	0.03	−2.52	0.012
Has Diabetic Retinopathy (ref = no)	−0.20	0.26	−0.76	0.446
Interaction between maximum HbA_1c_ and DR^b^	0.09	0.03	2.75	0.006

^*a*^ *Patients’ providers were modeled as a random effect to set apart characteristics attributed to providers rather than to patients*

^*b*^ *Denotes statistically significant effects associated with the number of visits at alpha = .05*

Responses from the survey on diabetes management goals, asked only of patients with two or more risk characteristics for the first 12 months of the program, are presented in Table 3. While 87.6% of patients surveyed were aware of their goal HbA_1c_, only 45.9% of patients knew the correct BP goal, consistent with findings by Duncan et al. [23].

**Table 3:** Diabetes Goals Survey Results (July 1, 2016-July 1, 2017)

	*Within the Subsample: risk score* *>* *2*	
	*n = 418 (patients who answered)*	
*Survey Questions and Responses*	*n*	*%*
HbA1c goal (%):		
< 7.0 (correct)	366	87.56
7.0–9.0	32	7.66
> 9.0	0	0.00
No recommendation	3	0.72
No/unclear answer	17	4.07
Blood Pressure goal (mmHg)		
< 140/90 (correct)	192	45.93
< 120/80	201	48.09
< 160/100	8	1.91
No recommendation	2	0.48
No/unclear answer	15	3.59

Patients (*N* = 1014) with two or more risk characteristics who were willing to extend their clinic visit to discuss available diabetes-related resources were also asked about their own perceptions of barriers to better self-management of diabetes (Table 4). Patients were allowed to choose multiple responses to this question. The most commonly reported barrier was “it’s just not working”, reported by 47.1% of patients who answered the queries (*N* = 240).

**Table 4:** Patient Self-Reported Barriers to Better Control of Their Diabetes

	*Within the Subsample: risk score* *>* *2*	
	*n = 240 (patients who answered)*	
*Barriers* ^*a*^	*n*	*%*
It’s just not working	113	47.08
No barriers	67	27.92
Depression/anxiety	19	7.92
Forgetting medication	18	7.50
Cost of supplies	5	2.08
Transportation	5	2.08
To get supplies	5	2.08
Getting an appointment	4	1.67
Medication cost	2	0.83
To get medications	2	0.83

^*a*^ *Patients were allowed to specify more than 1 barrier*

## Discussion

Implementation of the CDRP allowed the creation of a longitudinal dataset on patients with diabetes presenting to a retina clinic. In addition, data gathered is stored in a structured format as part of the EHR, allowing access by the treating physician and future analysis in an encounter-specific way. To the best of our knowledge, this is the first structured quality improvement program that combines systemic data from the EHR and queries from patients in a retina clinic. The CDRP is unique not only because of its novelty, but because data acquisition was incorporated into the routine workflow of a busy retina clinic, with minimal disruption to care and in an easily accessible format, allowing for future adoption and expansion to other practices. The program, which is the first to longitudinally combine systemic data with ophthalmic findings and management plans, enables the identification of metabolic and patient-specific personal attributes that influence the risk of complications.

The demographics are as expected for this cohort that presents to a large academic medical center in a relatively affluent medium-sized metropolitan area. Interestingly, more than 40% of patients with diabetes seeking retina care do not have a diagnosis of DR based on encounter diagnosis codes, implying that their clinic visits were either for retinopathy screening or for other diagnoses. Additionally, only a small fraction reported a history of kidney disease or had microalbuminuria. However, given that this data relied in large part on patient reports, it is likely that this is underreported. The overall cohort has low average maximum HbA_1c_, relatively well-controlled BPs, and low mean number of visits; however, the large ranges and distributions of maximum HbA_1c_ values (4.3–15.7%, and 77.38% of patients have maximum HbA_1c_ ≤ 9%), maximum SBP (80–237 mmHg) and DBP (40–123 mmHg), and number of visits over 18 months (1 to 27) suggest that significant healthcare resources are being devoted to a relatively small number of patients. In fact, only 35.58% of patients with DR (*N* = 1664) had any invasive treatment (including intravitreal injections and laser) during the 18-month study period (implying that others had mild or stable disease). These data show that even in a population skewed towards higher rates of DR and DR complications (as all patients in the cohort are persons with diabetes presenting to a retina clinic), a small number of patients require a large amount of health care resources. Our hypothesis that the distribution of clinical resources is skewed towards a small number of patients is further supported by the fact that increasing numbers of visits are correlated with duration of diabetes ≥10 years, and higher SBP and DBP. This is, to our knowledge, the first report to link systemic diabetes parameters to outcomes such as frequency of ophthalmology follow up, which is an important marker of cost of care. Furthermore, data from the CDRP were recently analyzed to show that BP is also significantly associated with receiving an intravitreal injection in patients with diabetes whereas HbA_1c_ is not [24]. This is also a previously unreported finding, and could have significant implications for ophthalmologists counseling patients with DR, and for the management of their retinal disease. Programs like CDRP may be valuable to identify which individuals require the most intensive treatment, and what factors led to their inclusion in that group.

Patients’ awareness of diabetes management goals was very good overall, with more than 87% of patients surveyed knowing their HbA_1c_ goal. Aiello and colleagues recently concluded that personalized risk assessment and education during ophthalmology visits did not improve glycemic control [25]. In that study, patients were provided details on risk assessment and scripted education by an ophthalmologist. Our findings suggest an explanation--that patients are already aware of their goal glycemic control, and reminders regarding this are likely unproductive.

In contrast, less than 46% of patients surveyed knew their BP goal, with approximately 48% overall overestimating the recommended degree of BP control. This data demonstrates that there is a disconnect between patients’ knowledge of appropriate HbA1c and BP goals and their ability to achieve them, a finding corroborated in much of the diabetes education literature [25–28].

A study conducted in 2017 showed that a greater level of perceived barriers to diabetes self-management is associated with close to 2.5 times greater odds of both having any DR and having severe DR [29]. Analysis of patients’ barriers to better management of their diabetes in our study highlights the difficulty associated with helping patients improve their diabetes management. The most common barrier reported by patients in the subgroup was that their efforts in self-management were “just not working,” (47.1%) followed by “no barriers” (27.9%). “Depression/anxiety” (7.9%) and “forgetting to take medication” (7.5%) were less prevalent barriers, but they are still important issues for caregivers to address. A recent study showed divergent perceptions of barriers to DR screening exams between providers and patients; financial burdens and depression were most commonly reported by patients as barriers to receiving screening, whereas providers did not rate these barriers as important [30]. It is possible that similar discrepancies between patient and physician perceptions of barriers to better self-management exist in the management of diabetes and its complications. As such, a more thorough understanding of perceived difficulties to self-management may be a key component to achieving better systemic disease control and may have potential long-term benefits to patients and health system resources.

A notable strength of this study is the large patient cohort. Continued analysis of the data has already identified possible risk factors contributing to the need for intensive treatment and possibility of vision loss [24]. With continued data collection and analysis, CDRP will also allow for development of benchmarks to determine quality of care. Future development of a “risk score” could lead to better prognostication and allocation of health system resources.

A limitation of this study is that as a retrospective analysis it is subject to biases, such as the inability to control for confounding factors not available from the patients’ EHR or surveys. Both assessment of baseline parameters and acquisition of survey data regarding patient barriers and knowledge of diabetes management goals depended on patients’ willingness to either get testing not usually done in an eye care setting (point of care HbA_1c_) or to spend extra time during their clinic appointment to provide this information (the barriers and diabetes goals surveys), which may cause selection bias in our analysis. Some of the data, most notably questions on history of kidney disease and duration of diabetes, were also subject to recall bias. Finally, patients may have reasons to avoid honestly reporting information, such as smoking status. The program’s ongoing nature, though, allows for adjustment to workflow and data collection techniques. Recent changes in data collection involve querying patients during their electronic check-in procedure, which reduces respondent burden and likely will increase response rate, as patients will no longer be required to prolong their clinic visit in order to provide information.

## Conclusions

Approximately 15–17% of blindness in the developed areas of the Americas and Europe can be attributed to DR [2]. Not only is DR a major cause of visual decline, but it also represents a significant financial burden to society both in health care costs and disability implications. These costs are only expected to rise in the near future. As such, programs like CDRP, which provide a better understanding of the characteristics of patients with diabetic eye disease, are essential to improving our ability to preserve visual function and to limiting strain on health system resources. This report describes the population of patients with diabetes presenting to a large academic retina practice and demonstrates how structured systemic data can be collected during routine eye care visits as well as the potential value—both to the patient and the health care system--of analyzing this data.

## Abbreviations

ADA: American Diabetes Association
BMI: Body mass index
BP: Blood pressure
CDRP: Comprehensive Diabetic Retinopathy Program
DBP: Diastolic blood pressure
DR: Diabetic retinopathy
EHR: Electronic health record
HbA_1c_: Hemoglobin A_1c_
SBP: Systolic blood pressure
